# Supplementary material for: An intervention to improve teacher well-being support and training to support students in UK high schools (the WISE study): A cluster randomised controlled trial
Source: PLoS Med. 2021 Nov 11;18(11):e1003847. doi: 10.1371/journal.pmed.1003847 (PMC8629387; doi:10.1371/journal.pmed.1003847)
Supplement: S1 Text — Table A. Results from imputed models for teacher WEMWBS outcome. Table B. Results from imputed models for teacher PHQ-8 outcome. Table C. Results from imputed models for student WEMWBS outcome. Table D. Results from imputed models for student SDQ outcome. (DOCX) [file pmed.1003847.s001.docx]

**S1 Text**

**Supplementary material: results from imputed models**

**Table A. Results from imputed models for teacher WEMWBS outcome**

|  | **MNAR rescaling parameters** | **Adjusted difference in means (95% CI)^a^** | **p-value** |
| --- | --- | --- | --- |
| **Teacher WEMWBS** | 1.00,1.00 | -0.79 (-1.96, 0.39) | 0.190 |
|  |  |  |  |
|  | 1.00, 0.95 | -1.75 (-2.96, -0.53) | 0.005 |
|  | 0.95, 1.00 | 0.08 (-1.10, 1.25) | 0.899 |
|  | 0.95, 0.95 | -0.88 (-2.09, 0.32) | 0.152 |
|  | 0.95, 0.90 | -1.84 (-3.09, -0.59) | 0.004 |
|  | 0.90, 0.95 | -0.02 (-1.23, 1.19) | 0.973 |
|  | 0.90, 0.90 | -0.98 (-2.23, 0.27) | 0.125 |
|  |  |  |  |
|  | 1.00, 1.05 | 0.17 (-0.98, 1.33) | 0.767 |
|  | 1.05, 1.00 | -1.65 (-2.83, -0.46) | 0.006 |
|  | 1.05, 1.05 | -0.69 (-1.85, 0.47) | 0.246 |
|  | 1.05, 1.10 | 0.28 (-0.88, 1.43) | 0.639 |
|  | 1.10, 1.05 | -1.55 (-2.72, -0.37) | 0.010 |
|  | 1.10, 1.10 | -0.59 (-1.75, 0.57) | 0.322 |

*^a^Fully adjusted models adjusted for region, FSM, gender and years of experience*

**Table B. Results from imputed models for teacher PHQ-8 outcome**

|  | **MNAR rescaling parameters** | **Ratio of geometric means^a^ intervention / control (95% CI)^b^** | **p-value** |
| --- | --- | --- | --- |
| **Teacher PHQ-8** | 1.00, 1.00 | 1.00 (0.91, 1.10) | 0.961 |
|  |  |  |  |
|  | 1.00, 0.95 | 0.97 (0.88, 1.06) | 0.494 |
|  | 0.95, 1.00 | 1.03 (0.94, 1.13) | 0.478 |
|  | 0.95, 0.95 | 1.00 (0.91, 1.09) | 0.974 |
|  | 0.95, 0.90 | 0.96 (0.88, 1.06) | 0.431 |
|  | 0.90, 0.95 | 1.03 (0.94, 1.13) | 0.518 |
|  | 0.90, 0.90 | 0.99 (0.91, 1.09) | 0.905 |
|  |  |  |  |
|  | 1.00, 1.05 | 1.04 (0.94, 1.14) | 0.448 |
|  | 1.05, 1.00 | 0.97 (0.88, 1.07) | 0.556 |
|  | 1.05, 1.05 | 1.01 (0.91, 1.11) | 0.902 |
|  | 1.05, 1.10 | 1.04 (0.94, 1.15) | 0.424 |
|  | 1.10, 1.05 | 0.98 (0.88, 1.08) | 0.624 |
|  | 1.10, 1.10 | 1.01 (0.91, 1.12) | 0.849 |

*^a^Ratio of geometric means reported due to violation of model assumptions*

*^b^Adjusted for region, FSM, gender and years of experience*

**Table C. Results from imputed models for student WEMWBS outcome**

|  | **MNAR rescaling parameters** | **Adjusted difference in means (95% CI)^a^** | **p-value** |
| --- | --- | --- | --- |
| **Student WEMWBS** | 1.00,1.00 | -0.04 (-1.17, 1.09) | 0.946 |
|  |  |  |  |
|  | 1.00, 0.95 | -0.43 (-1.55, 0.70) | 0.456 |
|  | 0.95, 1.00 | 0.29 (-0.83, 1.41) | 0.613 |
|  | 0.95, 0.95 | -0.10 (-1.21, 1.01) | 0.861 |
|  | 0.95, 0.90 | -0.48 (-1.60, 0.63) | 0.394 |
|  | 0.90, 0.95 | 0.23 (-0.88, 1.33) | 0.684 |
|  | 0.90, 0.90 | -0.16 (-1.27, 0.95) | 0.783 |
|  |  |  |  |
|  | 1.00, 1.05 | 0.35 (-0.80, 1.50) | 0.549 |
|  | 1.05, 1.00 | -0.37 (-1.52, 0.78) | 0.530 |
|  | 1.05, 1.05 | 0.02 (-1.15, 1.19) | 0.969 |
|  | 1.05, 1.10 | 0.42 (-0.78, 1.62) | 0.494 |
|  | 1.10, 1.05 | -0.31 (-1.50, 0.89) | 0.613 |
|  | 1.10, 1.10 | 0.09 (-1.14, 1.31) | 0.888 |

^a^Adjusted for region, FSM, gender and ethnicity

**Table D. Results from imputed models for student SDQ outcome**

|  | **MNAR rescaling parameters** | **Adjusted difference in means (95% CI)^a^** | **p-value** |
| --- | --- | --- | --- |
| **Student SDQ** | 1.00,1.00 | 0.06 (-0.56, 0.67) | 0.859 |
|  |  |  |  |
|  | 1.00, 0.95 | -0.08 (-0.68, 0.52) | 0.800 |
|  | 0.95, 1.00 | 0.17 (-0.45, 0.78) | 0.589 |
|  | 0.95, 0.95 | 0.04 (-0.56, 0.63) | 0.907 |
|  | 0.95, 0.90 | -0.10 (-0.68, 0.48) | 0.739 |
|  | 0.90, 0.95 | 0.15 (-0.45, 0.74) | 0.622 |
|  | 0.90, 0.90 | 0.02 (-0.56, 0.59) | 0.959 |
|  |  |  |  |
|  | 1.00, 1.05 | 0.19 (-0.45, 0.83) | 0.562 |
|  | 1.05, 1.00 | -0.06 (-0.68, 0.57) | 0.859 |
|  | 1.05, 1.05 | 0.08 (-0.57, 0.72) | 0.816 |
|  | 1.05, 1.10 | 0.21 (-0.48, 0.87) | 0.540 |
|  | 1.10, 1.05 | -0.04 (-0.69, 0.61) | 0.914 |
|  | 1.10, 1.10 | 0.10 (-0.58, 0.77) | 0.780 |

^a^Adjusted for region, FSM, gender and ethnicity
