# Supplementary material for: An intervention to improve teacher well-being support and training to support students in UK high schools (the WISE study): A cluster randomised controlled trial
Source: PLoS Med. 2021 Nov 11;18(11):e1003847. doi: 10.1371/journal.pmed.1003847 (PMC8629387; doi:10.1371/journal.pmed.1003847)
Supplement: Attachment — Submitted filename: response to reviewers.docx [file pmed.1003847.s003.docx]

| Many thanks to the five reviewers for their thoughtful comments and questions. We very much appreciated the positive comments about the quality of the paper and the trial itself. We have responded to each suggestion for improvement in turn in this table. | |
| --- | --- |
| **Editors requests**  1. Please include the study setting in the title before the colon. | We have added ‘UK high schools’ before the colon |
| 2. The Data Availability Statement (DAS) requires revision: If the data are not freely available, please include an appropriate email address for inquiries (this cannot be a study author). | This has been updated – the data are now published and open access, but we are awaiting the doi number to add to the statement |
| 3. Please add author summary | This has been added following the Abstract |
| 4a. Please report your abstract according to CONSORT for abstracts, following the PLOS Medicine abstract structure (Background, Methods and Findings, Conclusions) <http://www.consort-statement.org/extensions?ContentWidgetId=562> 4b.       Please combine the Methods and Findings sections into one section, “Methods and findings”. | We have combined Methods and findings into one section |
| 4c. Please ensure that all numbers presented in the abstract are present and identical to numbers presented in the main manuscript text. | We have checked and all numbers are identical to those in the main manuscript |
| 4d. Please quantify the main results (with 95% CIs and p values). | We have added p values |
| 4e. Please include the actual amounts or percentages of all relevant outcomes, not just coefficients. | We have added relevant amounts at T2 for each group along with the coefficients |
| 4f. Please include a summary of adverse events if these were assessed in the study. | These were not formally assessed as this was not a drugs trial and no adverse events were anticipated |
| 4g. In the last sentence of the Abstract Methods and Findings section, please describe the main limitation(s) of the study's methodology. | We have added the following sentence: “The study’s main limitations were a lack of blinding of research participants, and the self-report nature of the outcome measures.” |
| 4h. Please mention specific study implications without overreaching what can be concluded from the data; the phrase "In this study, we observed ..." may be useful | We amended the first sentence of the conclusions to include the suggested phrase |
| 5. At this stage, we ask that you include a short, non-technical Author Summary of your research to make findings accessible to a wide audience | We have added this |
| 6. Please replace the term “cost-effectiveness” with “cost evaluation” or similar as no formal cost-effectiveness analysis was done. | We have amended this in the two places where we referred to cost-ffectiveness; the abstract and the introduction |
| 7. Please complete the CONSORT checklist and ensure that all components of CONSORT are present in the manuscript, including [how randomization was performed, allocation concealment, blinding of intervention, definition of lost to follow-up, power statement]. When completing the checklist, please use section and paragraph numbers, rather than page numbers | This has been completed |
| 8. Please provide 95% CIs and p values for all estimates in the text and tables. | All 95% CIs and p values already in the tables. We have tidied up references in the text so they include all this information formatted in the same way |
| 9. For adjusted analyses, please also provide the unadjusted analyses in the text and tables. | We have added unadjusted analyses into tables 2 and 3 and the text in the results section. |
| 10. In the Discussion please talk about what the study adds to existing research and where and why the results may differ from previous research in similar settings; implications and next steps for research, clinical practice, and/or public policy; one-paragraph conclusion. | We have re-written the discussion to include fuller consideration of what the findings contribute to knowledge. We have also added a one-paragraph conclusion summarizing what the study adds, how it builds on/differs from previous findings, and next steps for research and policy. |
| 11. Please use the "Vancouver" style for reference formatting and see our website for other reference guidelines.  a)      Please ensure that journal name abbreviations match those found in the National Center for Biotechnology Information (NCBI) databases, and are appropriately formatted andcapitalized. <https://journals.plos.org/plosmedicine/s/submission-guidelines#loc-references>. Please ensure that weblinks are current and accessible to date. b)      Six names should precede et al, in all your references. c)      Please include access dates for all weblinks and ensure that all weblinks are current and accessible. | Vancouver style has been used. References have been checked for journal name and that six names have been used. Access dates for all weblinks included, and weblinks are all current and accessible. |
| 12. Please include line numbers in your next draft | These have been added |
| **Reviewer 1**  The  title  is not adjusted  with the procedure (  It the  title , the  students has not entitled but the  authors are mentioned about students in the  Methodology). It would rather to   write  the  students  in the  title? Whether  the   teacher  wellbeing support   affects the   mental health of students. Please  clarify the  students role in the  title. while the  authors  assess and  studies the  teacher wellbeing support on students | We have referred to students in the title and short title now |
| **Reviewer 2**  1.  Abstract - given that it's a prospective trial, I would recommend p-values are provided in addition to the confidence intervals, especially for the primary endpoint. | We have now added these |
| 2.  Page 5 - in the randomisation process, was a block randomisation used within strata? | Yes block randomization was undertaken, we have added this detail on page 8 |
| 3.      Page 9 "To explore whether perceived support to staff and students, and quality of staff-student relationships might explain any differences in outcome by arm" I would recommend rewording this slightly, as it currently implies a mediation analysis. | Wording has been changed to say: “To explore whether the hypothesised mechanisms of change - perceived support to staff and students, and quality of staff-student relationships - differed by intervention arm at follow up” (page 13) |
| 4. Page 9 "some strata were merged during recruitment due to the limited number of schools available" - can more information on this be provided, is this referring to the randomisation? I had thought there were only two strata (location and FSM level)? | Apologies this was not worded clearly, we have changed this to say: “some levels within the FSM strata were merged during recruitment due to the limited number of schools available (specifically medium and high were merged for the English sampling frame and medium and low were merged for the Welsh sampling frame)” page 13 |
| 5. Page 10 "(data available as supplementary material)" it may be the authors meant this could be added as supplementary material if necessary. I would recommend it is added. If it meant otherwise then I could not see supplementary material with the submission. | Our apologies for omitting to include this supplementary material, we will upload it with the revised manuscript |
| 6. I would recommend that the estimated ICC is reported, at least for the primary outcome, as it would help future research. | The ICC for the primary outcome has been added to the results section page 14. |
| **Reviewer 3**  1. I suggest that you specify in about a sentence how the geographical locations were selected and why. | We have added the following sentence on page 7: “These areas were selected due to proximity to the research team, but provided a wide range of schools in terms of academic attainment, inspectorate rating and socioeconomic catchment areas.” |
| 2. I would appreciate a bit more detail on how you ensured equal distribution of schools to the two variables (location and income) in the two study arms. | We have added the following text on page 8: “This sampling procedure resulted in two recruited schools within each location (England city/non city / South Central Wales / South East Wales) and FSM stratum (low / medium / high) combination. Within each of these pairings, one school was randomly allocated to each study arm.” |
| 3. I suggest the authors clearly separate the primary outcomes from the secondary outcomes. It's not clear on the onset if all the listed outcomes are considered primary or not according to the way they are laid out in this section here. | We have added two subheadings in the methods under outcomes: primary outcome and secondary outcomes to make this clearer. We have also added process outcomes as a third heading here. |
| **Reviewer 4** | Many thanks for your review - no changes required |
| **Reviewer 5**  1. The Methods section of the abstract should provide more information about the cluster trial (e.g., allocation ratio, number of clusters, number of participants, stratification variables, etc.) Secondary outcomes are not clear. Include the p values for the differences between the two arms and the regression models used for the analyses. | In the original submission we showed the allocation ratio by showing numbers of clusters in each study arm, and we included the overall number of clusters (25) in the findings section of the abstract. This information is now in the restructured methods and findings section of the abstract. We also included stratification variables and listed the secondary variables in the original submission. We have kept these in the revised version in the methods and findings section of the abstract. We have added p values to the abstract. |
| 2. Evidence in the introduction section is a bit outdated and irrelevant. Caldwell and others' systematic review, for example, is focused on universal and targeted interventions for children and adolescents in schools. It might be worthwhile for the authors to review the evidence on interventions for teachers (Yamaguchi et al 2020). Both Bonnell et al and Ford et al focus on whole-school interventions rather than teacher-targeted interventions. They have a few components that focus on teacher training. There is no clear connection between intervention mechanisms and outcomes in the logic model (Fig. 1). Provide more current evidence with links between intervention mechanisms and outcomes. Who were the healthy school coordinators and the CAMHS link workers? What was their role in implementing the intervention? A detailed profile of this human resource's background is needed. What was the intervention of the control arm? | We included the reference to Caldwell et al. (from 2019) because it sets the scene regarding school-based interventions, but we have added another reference – Goldberg et al., 2019, as this includes a whole-school focus and so is perhaps even more relevant here. We thank the reviewer for the suggestion of an article by Yamaguchi et al, 2020, but we cannot find this reference. We did find some references containing an author of this name, but these are all concerned with training teachers in content delivery in the classroom. The WISE intervention did not focus on classroom teaching. Rather, it was about training teachers in mental health literacy to improve support and relationships with students, which is why we have included references relevant to this. We included the Bonell (2018) and Ford (2019) trials because they not only had elements of teacher training but also elements focused on strengthening relationships, both of which have relevance to the WISE intervention. The Ford trial was teacher-targeted and not whole-school; it involved training a small number of teachers in behaviour management in each school. As the reviewer notes, the Bonell trial was whole-school, but that is still relevant as the intention was for the WISE intervention to create change at a whole-school level relating to mental health support and teacher-student relationships. The WISE intervention was not only teacher training; an equally key part was the provision of teacher peer support services in each school. Although to the best of our knowledge there are no RCTs reporting interventions to support teacher mental health, we have added one other reference – Naghieh et al., 2015 - which is a review of four interventions that aimed to increase support for teacher mental health.  We thank the reviewer for the helpful point that we could have included more discussion about the healthy school coordinators and CAMHS link workers, mentioned in the logic model. The paper already explains in the methods that the Healthy Schools Coordinators (HSCs) are employed by Public Health Wales and delivered the MHFA training. But we have added on page 9: “We planned for the HSCs and school link workers based within Child and Adolescent Mental Health Services (CAMHS) to provide ongoing support to the peer support services once they were set up.” Unfortunately this did not happen as planned, so we have also added the following as a limitation in the discussion: “Finally, although we planned to work with school link workers based within Child and Adolescent Mental Health Services (CAMHS) in England and with the Healthy Schools Coordinators (HSCs) in Wales to provide ongoing support to the peer supporters, this did not happen in practice. In England this was because the school link worker role was not filled for many of our study schools, and in Wales this was because the HSCs who had the capacity to take on the trainer roles were not those who were supporting the study schools. This limitation is unlikely to explain the study’s main finding that overall teacher wellbeing did not improve, but it may have contributed to the peer support services losing some sense of momentum and being fully embedded in school life, as discussed below.”  The control arm did not have a specific intervention, as we noted in the previous version schools in the comparison group continued with usual practice. What this means varies considerably from one school to another, so we have added the following sentence to make this clearer (page 10): “In England and Wales there is no standard training package for teachers regarding mental health support, although schools are free to choose to pay for any such training that is available. Similarly there is no standard mental health support available to staff, although schools are free to buy such a service in, for example there are several Employee Assistance Programmes (EAP) available in the UK for workplaces.” |
| 3. There is also no information provided on the acceptability and feasibility of the intervention and its adaptation to the school setting. The latter is briefly discussed as one of the limitations of the study. | We examined acceptability and feasibility in an earlier pilot intervention, referenced in this paper. We have added a brief summary of our findings from this pilot regarding acceptability and feasibility in the following text on page 6: “We tested the acceptability and feasibility of the intervention in a pilot study of six schools, through observations of the training, interviews / focus groups with training attendees and the wider staff body, and surveys of staff and students.^11^ This pilot study found that schools and staff within schools were willing and able to participate in the intervention, that the MHFA training was perceived to be appropriate for the British secondary school context, and that it improved mental health knowledge and attitudes, and that the peer support service was feasible to establish, and perceived to be helpful. Barriers to the success of the peer support service were identified which we addressed in this main study. Specifically we strengthened the guidance provided to peer support services relating to advertising and confidentiality procedures, we increased the number of peer supporters in each school, we extended the length of intervention time by one year, and we emphasised the importance of senior leadership support. We also added shorter training component delivered to the whole teaching team, which included information about the peer support services.” As the intervention (the peer support plus training components) was designed specifically for the school setting, we cannot comment on adapting it to the school setting. |
| 4. There is a need for supportive literature for the second hypothesis. How improving the mental health and wellbeing of teachers can improve relationships between teachers and students and hence result in better mental health for students?  The logical framework fails to recognize the complex relationships between various levels of outcomes. The purpose of clubbing process outcomes with outcomes is not clear. As described in the hypothesis, a positive student-teacher relationship may improve student mental health as a result of better mental health of teachers. | We have added two references citing evidence regarding the relationship between teacher mental health and quality of relationships with students: Hoglund et al., 2015 and Sisask et al., 2014.  We hope that by having separate headings for outcomes and process outcomes the distinction between the two is clear. We decided it was important to include in the outcomes section of the logic model the measures that we would use to assess process outcomes as well as final outcomes.  We agree in hindsight that the logic model could more clearly capture the iterative association between teacher and student mental health, possibly with the addition of more arrows. However, it does not seem appropriate to retro-change the logic model at this point, as this was the one we developed following our pilot study that underpinned this study. We hope we have discussed these links between teacher and student mental health via supportive relationships sufficiently in the text. |
| 5. More details on the trial design are required. How many schools were randomized to each arm? What was the distribution based on two geographic locations? What was the randomization method? What are 'mainstream' and 'alternative provision' secondary schools? What do low, medium, and high levels of FSM mean? | We have added these details about number of schools in each arm and by geographical location and about the randomization method on page 8. These details are reported thus: “Following discussion about the possibility of school drop out, we randomly selected one additional school for participation from the English sampling frame. In total there were 12 participating schools in Wales, and 13 participating schools in England.  This sampling procedure resulted in two recruited schools within each location (England city/England non city / South Central Wales / South East Wales) and FSM stratum (low / medium / high) combination. Within each of these pairings, one school was randomly allocated to each study arm. Block randomisation was undertaken by the study statistician, who was blinded to the schools’ identities, using the Stata *ralloc* command. This process resulted in 12 intervention schools (6 in Wales and 6 in England) and 13 comparison schools (6 in Wales and 7 in England). Random allocation to study arm took place after baseline data collection.”  We have added details about what mainstream schools and alternative provision means on page 7: “regular schools for students aged 11-18 where individuals with and without special educational needs are taught together” and “provision for students unable to attend mainstream school, for example due to learning or physical disability or because they have been excluded from mainstream education”. We have added detail to our definition of low, medium and high levels of FSM on page 8: “high, medium and low proportion of students eligible for free school meals compared to the national average”. |
| 6. Were there any inclusion and exclusion criteria for students? | There were no inclusion or exclusion criteria for students beyond what is stated regarding year group and consents. The following statement has been added on page 7: “There were no inclusion or exclusion criteria for teachers or students in year 8. At the follow up time points a small number were ineligible to receive the questionnaire, due to having left the school, or being absent from school long-term (for example due to long-term sickness).” |
| 7. How was the opt-out assent procedure employed for teachers and students? | We have added the information on page 8: “instructions stated that those who did not wish to complete the questionnaire should place the blank copy into the envelope provided and return to the study team.” |
| 8. Were the student assessments also paper-based? Who administered the questionnaires to the teachers and students? Were teachers present when the student assessment carried out in the tutor groups/lesson time? Were the data collectors masked to the school's allocation status? If yes/no, reasons for it. | Yes the student assessments were paper-based, we have added the word “paper” on page 9 to make this clear. We have also added details regarding the process when students were absent, who conducted the data collections and whether teachers were present for the student data collections on page 9: All data collections were conducted in school, led by a study team member, accompanied by trained fieldworkers. Individual level teacher outcomes were collected through self-report paper questionnaires during staff meetings in May-July 2016 (T0, pre-intervention), May-July 2017 (T1, 1 year follow-up) and May-July 2018 (T2, 2 year follow-up). Those who were absent were offered the option of completion via an online survey. Individual level student outcomes were gathered via self-report paper questionnaires at T0 and T2 during tutor groups or lesson time. Where less than five students in a tutor group were absent the paper questionnaires were left for them to complete when next in school, under the supervision of school staff. Where more than five were absent a second data collection session administered by a study team member was arranged. At least one teacher was present in the room during student data collections to adhere to school child protection policies and assist with behaviour management. They were given a written information sheet explain the data collection process, and requesting that they did not look at the students’ questionnaires, or involve themselves with the data collections.”  We have added details about whether the data collectors were blinded on page 9: “Schools and the study team including those who collected the questionnaire data were not blind to allocation at the follow up data collections. In the case of school staff, this was because it was not possible to conceal the intervention delivery from them. In the case of the data collections, these staff had also been the ones to organise the intervention delivery due to resource limitations.” |
| 9. Which were the primary outcome(s) and secondary outcomes? | Apologies if this was not clear enough, we have added headings indicating primary, secondary and process outcomes in the methods section. |
| 10. Which version of the WEMWBS was used? Please provide further details on WEMWBS items, scoring, and validity of WEMWBS in the adult target population. | WEMWBS only has one version; we have added the detail that we used the 14-item scale (as opposed to the SWEMWBS which is 7 item). We have added these other details on page 10: “The WEMWBS incorporates elements of both subjective and psychological wellbeing, represented in fourteen statements such as ‘I’ve been feeling cheerful’ and ‘I’ve been feeling interested in other people’. For each statement, respondents select one out of five options: none of the time/rarely/some of the time/often/all of the time. Each response is scored from 1 (none of the time) to 5 (all of the time), and the total is summed to make up a wellbeing score of between 14 and 70, where higher scores represent better wellbeing. The scale has been validated in the general UK adult population, with a Cronbach’s alpha score of 0.91, and test-retest reliability at one week of 0.83.^21^” |
| 11. Although the authors mentioned that the details on intervention are published elsewhere, some details on the intervention mechanism, broad topics, and strategies of training, intervention active duration, support and supervision in offering peer support service and establishing further services for students, etc. are needed. | We have added a figure – figure 2 – which summarizes more detail about the intervention. |
| 12. Clarification on which version of the SDQ was used is required. | We have added that this was the short form version without the impact assessment |
| 13. How were the items related to perceived support and relationships were scored? What were the possible minimum and maximum scores? | These items were all separate as opposed to making up a scale with possible minimum and maximum scores. We have added this information on page 11: “The teacher survey contained five questions and the student survey contained three items, all of which were separate items as opposed to items on one scale.”  We have added further information again clarifying these are single items on page 13 where we describe the analysis: “To explore whether the hypothesised mechanisms of change - perceived support to staff and students, and quality of staff-student relationships - differed by intervention arm at follow up, all the process evaluation single items were recoded into binary variables (agree/disagree). Logistic regression models (one per item) were used to compare outcomes for these variables between arms at T2, adjusted for T0 scores, school-level FSM and geographical area.” |
| 14. Details on the process evaluation are required (e.g., frequency, data collection method, who collected process data, indicators selected, etc.) | The process evaluation outcomes were collected in the same surveys, and therefore via the same procedures, as the main outcomes. Apologies that this was not clear enough; on page X, we have added the information: “Process outcomes were collected in the same teacher and student surveys as the outcome measures.” |
| 15. Economic evaluation: it is not appropriate to call the cost-estimation exercise a cost-effectiveness evaluation. | We have changed this to say “cost evaluation” in the abstract |
| 16. Did the authors account for loss to follow-up in the power calculation? Were the assumptions for power calculation rooted in actual data? Provide the citation for ICC value. | Yes we accounted for loss to follow-up and our assumptions were rooted in actual data. We used findings from the pilot study to inform assumptions relating to response rates, sample sizes and the ICC. We have repeated the reference to the pilot study in this section of the methods, page X. We have also added the following information on page 12: “We assumed a mean of 50 teacher responses per school (with a coefficient of variation of sample size of 0.5) – which reflects the response rate of approximately 75% of teachers achieved in the pilot study^11^” |
| 17. Statistical analyses: Mention the way findings are reported. | We have added the following on page 13: "Findings are reported in line with CONSORT guidelines extension to cluster randomised trials” |
| 18. Why are the process evaluation results not presented? | We do present the process evaluation results (table 3) that relate to the second and fourth process outcomes in our logic model. Other mixed methods process evaluation results relating to implementation and perceptions of the intervention have already been reported – this paper is cited in this section on page 6, Fisher et al., 2020, and a summary of these findings is included in the discussion on page 18. There is one final paper being drafted that contains some further qualitative process findings, which will explore the conclusions presented here regarding the need to change the whole school context. We did not want to detract from the main aim of this paper – which is to report on the main outcomes - by including all the remaining process evaluation methods and findings too, which would double the paper’s length.  It is common for trials that included complex process evaluations not to publish both the main trial outcomes and the whole process evaluation in one paper. We have added text clarifying this division of reporting of the process evaluation outcomes on pages 6-7 . |
| 19. What was the ICC for the primary outcome at baseline? | We have added this on page 14, it was 0.021 (95% Cis 0.007, 0.062). |
| 20. What kinds of severe adverse events observed? | There were no severe adverse events – defined as an occurrence that results in death, is life-threatening, requires inpatient hospitalization, or results in persistent or significant disability or incapacity – recorded for any participants during the study. We have added this information on page 8 |
| 21. There is not enough and convincing discussion of no impact of the intervention on outcomes or mechanisms. The process evaluation results are not presented and discussed sufficiently to unpack the findings of the trial. | In table 3, we present the survey findings which measured process outcomes relating to teacher work related stress, perceptions of whole-school attitudes towards staff and student wellbeing, and quality of teacher-student relationships. These show no impact at a whole population level, as perceived by teachers and students, of the intervention on these hypothesised mechanisms of change. We also re-state these findings on page 16. We feel this is sufficient presentation of the process evaluation results relating to mechanisms of change. We have re-worked and expanded the discussion to unpack these findings in more detail, particularly from the bottom of page 17 onwards. |
| 22. Why do the authors consider the non-blinding of the participants as one of the limitations of the study? How would knowing the intervention allocation status influence the responses of teachers and students? | Lack of blinding as a risk factor for biased RCT results is well established. In the example of this study, we suggest in the discussion that, because the outcomes were self-report measures, knowledge of being part of an intervention may have influenced teachers responses in particular (students were less likely to have been aware they were part of an intervention). We have added a further sentence spelling out how this may have had an influence on outcomes, page 16: “For example, if participating in the intervention led to raised expectations regarding changes in school, and if these expectations were not perceived to be met, then teachers in intervention schools may have given more negative responses about their school environment or their own mental health and wellbeing at follow up.” |
| 23. The authors have discussed the school context as one of the limitations of the study. However, it should be presented as an explanation for receiving the negative findings among many other potential reasons. | Our intention was always to suggest context was an explanation for the null findings. We have hopefully made this clearer now in the expanded discussion, from page 17 onwards. |
| 24. In the absence of sufficient information on control arm intervention, it is difficult to interpret the findings. | As noted above, we have added information about usual practice in the methods. In the expanded discussion we have explicitly considered whether insufficient differentiation from usual practice was an explanation for the null findings. |
| 25. Minor: Spell out abbreviations when used for the first time (e.g., Page 3 WISE intervention) | We spell out the abbreviation WISE in the parentheses that follow (Wellbeing in Secondary Education), we presented it this way round as WISE is the name of the intervention, albeit an acronym. We have checked and for all other abbreviations we have spelled them out in full the first time they are used. |
